# Lactate Biology: Subcellular Routing and Chemical Form Define Function

**DOI:** 10.1021/acs.biochem.6c00251

**Published:** 2026-06-29

**Authors:** Nicholas A. Offei, Ahmad A. Cluntun

**Affiliations:** Department of Biochemistry and Molecular Biology, Rutgers Robert Wood Johnson Medical School, Rutgers, The State University of New Jersey, Piscataway, New Jersey 08854, United States

## Abstract

Lactate has undergone a major conceptual shift, from a glycolytic waste product to a circulating metabolic currency and, more recently, a multifunctional regulator of physiology. It serves as a mitochondrial fuel, an epigenetic modifier via protein lactylation, a ligand for the G-protein-coupled receptor HCAR1, and a precursor for endocrine-like N-lactoyl amino acids. This convergence raises a central question: how can a single metabolite support such distinct roles without functional conflict? We propose that the resolution lies not in lactate concentration alone but in two complementary organizing principles: subcellular routing and chemical form. Transporter localization, enzyme compartmentalization, donor formation, and metabolic competition bias lactate toward distinct biochemical fates, while conversion into chemically distinct intermediates—including lactyl-CoA, lactoyl-glutathione, d-lactate, and N-lactoyl amino acids—further constrains the biological outcomes that lactate can support. Lactate’s fate is influenced by the cellular compartments it accesses, a process constrained by specific monocarboxylate transporters at the plasma and mitochondrial membranes, isoform-specific localization of lactate dehydrogenases, and compartmentalized enzymatic machinery that converts lactate into distinct biochemical donors. Mitochondrial oxidation, protein lactylation, extracellular signaling, and N-lactoyl amino acid synthesis should therefore not be viewed as parallel consequences of elevated lactate concentration. Instead, they represent interconnected metabolic fates that draw from shared lactate pools and are influenced by compartmental access and local enzymatic context. Here, we integrate evidence from metabolism, epigenetics, and signaling into a spatial framework in which lactate function depends on where it is routed. In this view, lactate is not a promiscuous metabolite but a compartmentalized intermediate whose biological effects are shaped by spatial context. We further distinguish between established, emerging, and speculative aspects of this compartmentalized view to highlight key gaps and prioritize future experimental testing.

## Graphical abstract

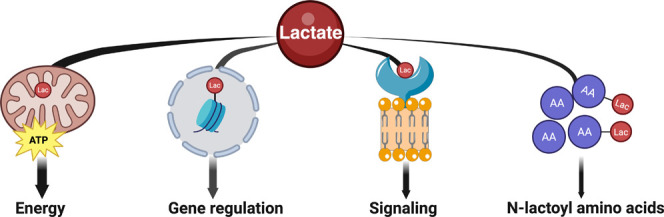

## 1 Introduction

Since Warburg’s early studies in the 1920s, lactate (2-hydroxypropanoate) has been regarded as the end product of anaerobic glycolysis, a metabolic waste generated when oxygen is limiting. This view persisted for nearly a century, reinforced by associations between lactate accumulation, hypoxia, and strenuous exercise. Yet this view overlooked a key observation: lactate is produced continuously, even under fully aerobic conditions. Among circulating metabolites in humans, lactate (∼1 mM at baseline, depending on physiological state) is second in abundance only to glucose (∼5 mM),^1^ reflecting its status as a basal metabolic intermediate generated by nearly every cell type. Its persistent abundance across metabolic states suggested a broader role in cellular physiology than previously appreciated. The first major transition in lactate biology came with George Brooks’ “lactate shuttle” hypothesis, which reframed lactate as a dynamic circulatory currency exchanged between tissues to sustain redox balance and energy homeostasis.^2–4^ A second conceptual shift is now underway. Lactate is recognized not only as a fuel but also as a signaling molecule, a precursor for post-translational modifications, and a substrate for a newly discovered class of endocrine metabolites. It activates the G-protein-coupled receptor hydroxycarboxylic acid receptor 1 (HCAR1);^5–8^ it serves as a substrate for lysine acyl modification on histones and nonhistone proteins;^9,10^ it is enzymatically conjugated to amino acids to form N-lactoyl-phenylalanine and related molecules that regulate feeding behavior;^11,12^ and it is oxidized directly within mitochondria via an inner membrane transport and oxidation system.^13,14^ Taken together, these discoveries create a central mechanistic challenge: how a single metabolite, whose functions have largely been studied independently, can execute such distinct functions without crosstalk, competition, or conflict (Table 1).

**Table 1:** Evidence Supporting Distinct Lactate Routing Pathways

lactate fate	compartment	key components	evidence level	key support	open questions	experimental tests
oxidation	mitochondria	MCT1, LDHB	emerging/context dependent	isotope tracing, respiration studies	tissue generality, regulation	compartment-targeted biosensors
signaling	extracellular	HCAR1	established	GPCR pharmacology, human infusion studies	lactate source discrimination	MCT4-specific lactate release tracking
lactylation	nuclear/cytosol	p300, GTPSCS	established mark; donor route emerging	mass spectrometry proteomics	dominant lactyl donor in vivo	nuclear lactate biosensors, lactyl-CoA quantification
N-lactoyl AAs	cytosol	CNDP2	emerging	exercise metabolomics, CNDP2 knockout	substrate mechanism, tissue distribution	CNDP2 inhibition during graded exercise
MG detox	cytosol	GLO1/2	established	biochemical characterization	link to signaling, Kce vs K-d-la	d-lactate tracing under MG stress

Many prevailing models have implicitly assumed that lactate freely equilibrates across cellular compartments and that its various functions are simultaneous consequences of elevated concentration. However, lactate’s major fates draw from shared pools and operate in different compartments: mitochondrial oxidation consumes lactate; lactylation incorporates lactate-derived carbon into stable protein modifications; HCAR1 signaling is triggered by extracellular lactate; and N-lactoyl amino acid synthesis diverts lactate into exportable metabolites. These processes can occur in parallel, but they still compete for lactate and are constrained by compartmental access and enzymatic context. They are not mutually exclusive but neither are they independent, as they draw from shared lactate pools separated by intracellular boundaries. Importantly, lactate does not act exclusively as free l-lactate. Several biologically relevant functions are mediated through chemically distinct derivatives, including lactyl-CoA, lactoyl-glutathione, d-lactate, and N-lactoyl amino acids. These species differ in reactivity, localization, and biological output. Thus, lactate biology may be governed by two orthogonal variables: where lactate is routed and what chemical form it assumes.

To address this, we propose a subcellular routing framework that shifts the focus from lactate concentration alone to the biased allocation of lactate toward distinct biochemical fates (Figure 1). Throughout this perspective, we define lactate routing as the biased allocation of lactate toward distinct biochemical fates through transporter localization, enzyme compartmentalization, donor formation, and metabolic competition. Some aspects of routing likely emerge passively from thermodynamics and enzyme localization, whereas others may involve regulated transport or compartment-specific donor generation. The central premise is that lactate trafficking is constrained at key cellular barriers. At the plasma membrane, monocarboxylate transporters (MCTs) with distinct affinities, expression patterns, and transport kinetics regulate lactate exchange, with MCT1 and MCT2 favoring import in oxidative cells^15–17^ and MCT4 favoring export from glycolytic cells.^18^ Across the mitochondrial inner membrane, a dedicated MCT1 import complex may support mitochondrial lactate oxidation.^13,14^ Although lactate is generally assumed to diffuse through nuclear pores, whether nuclear lactate utilization is governed solely by passive diffusion or additionally shaped by localized donor formation remains unresolved. Because evidence supporting lactate routing differs substantially across proposed pathways, this perspective distinguishes between established mechanisms (e.g., plasma membrane transport via monocarboxylate transporters), emerging evidence (e.g., mitochondrial lactate oxidation), and more speculative models (e.g., nuclear lactate routing and localized receptor signaling).

**Figure 1:**
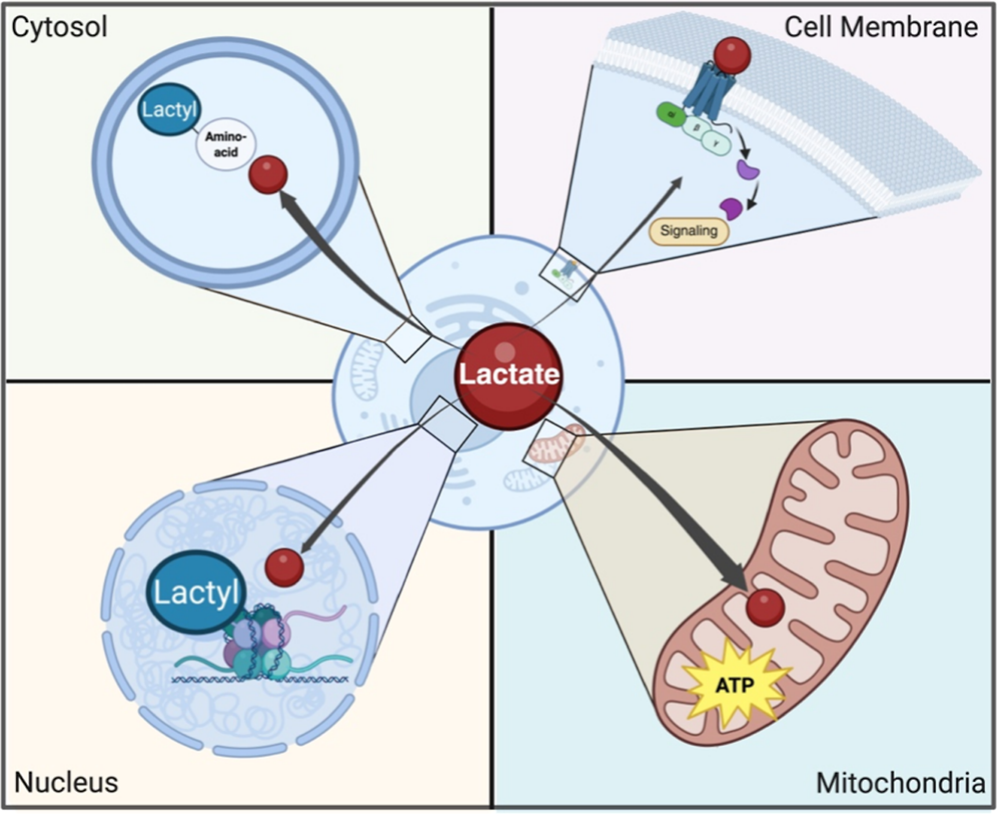
Subcellular routing determines lactate function. Schematic representation of the subcellular routing framework illustrating how lactate function is determined by subcellular destination rather than concentration alone. Lactate (red circle) is routed into four spatially distinct fates. In the cytosol, conjugation with amino acids generates N-lactoyl amino acids, defining a diversion fate. At the plasma membrane, extracellular lactate activates Gi-mediated signaling, constituting a signaling fate. In the nucleus, lactate-derived lactyl groups modify histone and nonhistone proteins, establishing a regulatory fate. Mitochondrial import enables oxidation and ATP production, specifying an energetic fate. These routes draw from shared lactate pools but are not mutually exclusive. Their relative engagement is shaped by metabolic state, compartmental access, and cellular demand. Arrow thickness is intended to conceptually illustrate differences in engagement among lactate fates and does not imply quantitative flux.

Compartmentalization is reinforced further by the localization of enzymes that consume or transform lactate. Lactate dehydrogenase (LDH) isoforms are not functionally redundant; LDHA is predominantly cytosolic and strongly favors pyruvate reduction to regenerate NAD^+^ for glycolysis, whereas LDHB is associated with mitochondrial compartments and preferentially supports lactate oxidation.^13,14,19^ Similarly, carnosine dipeptidase 2 (CNDP2), which generates N-lactoyl amino acids, resides in the cytosol;^11^ histone acetyltransferases that catalyze lactylation are nuclear;^20,21^ and the GTP-specific succinyl-CoA synthetase (GTPSCS) complex that synthesizes lactyl-CoA is nuclear-enriched.^20^ This spatial organization restricts specific reactions to defined compartments, limiting the indiscriminate crosstalk.

Finally, lactate-derived intermediates are spatially segregated and noninterchangeable. Lactyl-CoA, lactoyl-glutathione, and d-lactate arise from distinct precursor pools via different enzymatic (or nonenzymatic) mechanisms and access different target proteins or pathways. The dominant lactyl donor in vivo, therefore, likely depends on local metabolic conditions and donor accessibility within each compartment.

Drawing on evidence from transporter biology, mitochondrial metabolism, epigenetic regulation, GPCR signaling, and metabolomics, we propose a unified spatial model of lactate function in which distinct destinations define distinct biological outcomes. These outcomes are shaped by metabolic state, enzyme localization, and cellular context, including energy demand, redox balance, and signaling requirements, such that lactate utilization reflects the physiological context rather than passive accumulation. Viewing lactate through the lens of subcellular routing resolves several conceptual conflicts and generates directly testable hypotheses. More broadly, we propose a general principle of metabolic organization: for multifunctional metabolites, cellular address and chemical identity help to determine biological function.

## 2 Determinants of Lactate Routing

### 2.1 Plasma Membrane MCTs: Import and Export

At the plasma membrane, lactate flux is regulated by the specialized affinities of proton-coupled MCTs, primarily MCT1 and MCT4. These proteins belong to the 14-member SLC16 gene family, with MCTs 1–4 being the primary facilitators of proton-coupled lactate, pyruvate, and ketone body transport.^22^ Proper localization and function of these transporters depend on specific chaperones, such as CD147 and GP70. These chaperones are essential for quality control, trafficking, and stabilization of the transporters at the cell surface.^23,24^ MCT-mediated transport is not intrinsically directional; rather, flux is thermodynamically determined by local lactate and proton gradients.^18,25^ These transporters possess distinct kinetic properties that favor net flux toward import or export under physiological conditions. MCT1, with its high lactate affinity (*K*_m_ ∼3–5 mM), efficiently imports extracellular lactate into oxidative cells. Conversely, MCT4 has low affinity (*K*_m_ ∼22–28 mM) and high capacity, favoring efflux from glycolytic cells.^22,26,27^

These kinetic differences shape both transport direction and downstream metabolic fate. Lactate imported through MCT1 is typically taken up by oxidative or other nonglycolytic cells and routed toward fates distinct from lactate generated endogenously and exported through MCT4. Endogenous lactate exported via MCT4 is positioned for circulation, uptake by distant tissues, or engagement of extracellular HCAR1. Moreover, this functional specialization is reinforced by transcriptional programs. Hypoxia-inducible factor 1-α (HIF-1α), stabilized under hypoxia or by oncogenic signaling, coordinately upregulates glycolytic enzymes and MCT4, reinforcing the lactate-exporting phenotype.^28^ Peroxisome proliferator-activated receptor gamma coactivator 1-alpha (PGC-1α), a master regulator of oxidative metabolism, promotes MCT1 expression and mitochondrial biogenesis, creating demand for lactate as an oxidative substrate.^29^ Together, these transcriptional programs shape lactate availability and influence whether it is oxidized, exported, or diverted into nonoxidative pathways.

While the transcriptional upregulation of these transporters is a foundational mechanism for establishing lactate trafficking, an unresolved question is whether MCT localization is dynamically regulated beyond the transcriptional control. Can MCT1 be rapidly recruited to the plasma membrane from the intracellular pools? Can it be redirected to mitochondrial membranes? Does MCT4 ever function as an importer under specific conditions? These questions bear directly on whether lactate routing is acutely adjustable or primarily set by the differentiation state.

### 2.2 Mitochondrial MCT1 and the Lactate Oxidation Complex

A key line of evidence supporting compartmental control of lactate fate is the mitochondrial lactate oxidation complex (mLOC), originally proposed in the intracellular lactate shuttle (ILS) hypothesis.^30^ Early formulations suggested this possibility, but the mechanism remained unclear.^14^ More recent biochemical evidence supports a mitochondrial-associated pool of MCT1 and LDHB but not MCT4 or LDHA, within a proteinase-protected inner membrane compartment.^13,16^ This localization may help resolve a longstanding paradox: how cells can oxidize lactate without compromising cytosolic NAD^+^ availability for glycolysis. Whether this mechanism operates broadly across tissues or instead reflects a context-specific adaptation remains unresolved.

Cytosolic lactate oxidation consumes NAD^+^, competing directly with glycolysis and suppressing glycolytic flux. By sequestering lactate oxidation within the mitochondrial matrix, the cell couples lactate-derived NADH directly to the electron transport chain while preserving cytosolic NAD^+^ for glyceraldehyde-3-phosphate dehydrogenase. Thus, the mLOC may represent not merely an additional pathway but a compartmental solution to redox competition (Figure 2). MCT1’s proton-coupled symport mechanism may support mitochondrial lactate import without direct ATP expenditure.^31^ Once in the matrix, LDHB catalyzes lactate oxidation to pyruvate, generating NADH that feeds Complex I. In this context, lactate functions not merely as a glycolytic end-product but as a direct respiratory substrate.

**Figure 2:**
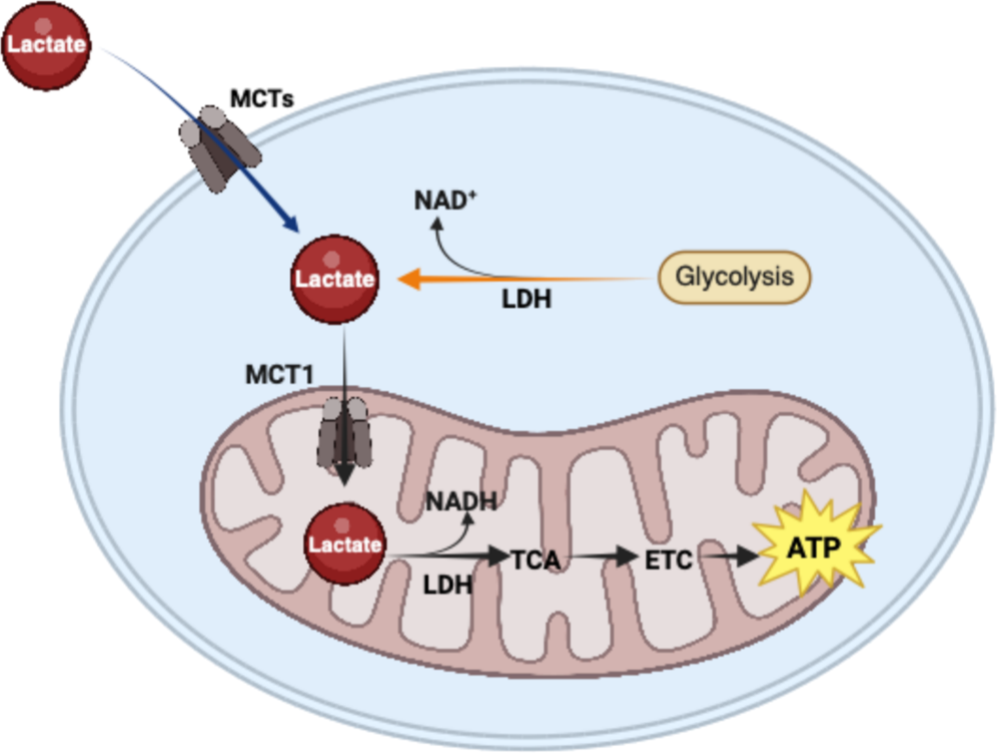
Mitochondrial routing of lactate resolves cytosolic redox competition.

Lactate enters the cell via plasma membrane MCTs and is imported into the mitochondrial matrix via MCT1. Inside the matrix, LDH-mediated oxidation generates NADH that feeds the electron transport chain with pyruvate entering the TCA cycle to produce ATP. In the cytosol, LDH-driven lactate production from glycolysis regenerates NAD^+^ to sustain the glycolytic flux. This spatial separation may minimize direct redox competition between mitochondrial lactate oxidation and NAD^+^-dependent glycolysis.

Beyond the transporter complex itself, lactate routing to oxidation is enforced by isoform-specific lactate dehydrogenase (LDH) machinery. LDH is the central enzyme regulating lactate-pyruvate interconversion, but its function is critically determined by isoform-specific kinetics and subcellular localization. The five LDH isoforms (LDH-A through LDH-E), though structurally similar, possess distinct kinetic properties that dictate metabolic flux.^32,33^ Crucially, LDHA (higher affinity for pyruvate; *K*_m_ pyruvate ∼0.26 mM) strongly favors pyruvate reduction to lactate (regenerating NAD^+^ for glycolysis), while LDHB (higher affinity for lactate; *K*_m_ pyruvate ∼0.17 mM) favors lactate oxidation.^34^ We propose that this metabolic partitioning couples lactate-derived reducing equivalents to mitochondrial respiration while preserving cytosolic redox balance.

This kinetic specialization is reinforced by compartment-specific localization. LDHA is predominantly cytosolic, where its high catalytic rate and favorable thermodynamics ensure rapid NAD^+^ regeneration to sustain glycolysis.^13,35–37^ In contrast, LDHB has been reported in mitochondrial-associated compartments and is enriched in oxidative tissues, supporting lactate oxidation in settings where the resulting reducing equivalents can be coupled to mitochondrial respiration. Thus, we propose that LDH isoform diversity is not simply a kinetic optimization but a spatial enforcement mechanism that prevents futile redox competition and preserves distinct metabolic identities.

This kinetic and compartmental localization explains classic physiological observations, such as the correlation between falling muscle pH, lactate accumulation, and a rising cytosolic NADH/NAD^+^ ratio during exhaustive exercise.^38^ The system is designed to prioritize the LDHA-driven, NAD^+^-regenerating reaction in the cytosol while outsourcing oxidation to the mitochondrially routed LDHB pathway. Together, isoform-specific kinetics and localization direct lactate flux while preventing redox competition between the production and oxidation.

Remaining questions center on the regulation. Is the mLOC expression constitutive or dynamically regulated? Does it require specialized assembly factors? Can its activity be modulated posttranslationally? And importantly, is mLOC activity impaired in diseases characterized by apparent lactate oxidation defects, such as some mitochondrial disorders or insulin-resistant states?

### 2.3 Nuclear Pores as an Unresolved Frontier

Nuclear lactate metabolism presents a conceptual challenge for the routing framework. Current evidence suggests that lactate can diffuse through nuclear pores,^39^ implying that nuclear access is not actively gated. Whether this assumption holds under all metabolic conditions, however, has not been tested.

Yet, this interpretation may be premature. First, nuclear pores are not passive sieves; they exhibit selective transport for molecules above ∼40 kDa,^40^ but small metabolites are generally assumed to equilibrate freely. Direct measurements of nuclear versus cytosolic lactate concentrations under varying metabolic conditions are surprisingly scarce. Second, even if lactate itself diffuses freely, the lactyl donor may not. The recent identification of a nuclear GTPSCS complex as a lactyl-CoA synthetase indicates specialized nuclear lactate metabolism^20^ and supports a model in which lactate enters the nucleus and is locally converted into activated donor species, rather than being transported in its CoA-bound form. Third, it remains possible that regulated transport of lactate or its precursors into the nucleus occurs via unidentified carriers.

Resolving whether nuclear lactylation depends primarily on bulk lactate concentration or on regulated donor formation is critical to determining whether this spatial organization applies uniformly across the compartments. If nuclear lactylation requires donor allocation, then the field must identify the transporters or channels that mediate this process. The answer will help determine how broadly this framework applies across the intracellular compartments.

Future work using compartment-targeted biosensors and nuclear isotope tracing will be essential for determining whether nuclear lactylation is driven primarily by bulk lactate concentration or by compartment-specific donor formation.

### 2.4 Lactate Origin as a Determinant of Functional Routing

The functional fate of lactate is biased at the point of entry into the cellular metabolic network. Cellular lactate pools arise primarily from glycolytic flux,^35,41–43^ glutaminolytic conversion,^44^ and extracellular uptake,^4^ each coupled to distinct enzyme systems that influence its downstream fate.

Glycolysis constitutes the universal cytosolic source of lactate, with continuous production even under aerobic conditions.^3^ The Warburg effect in cancer cells, classically interpreted as excess lactate accumulation, may instead reflect an extreme reprogramming of intracellular routing, prioritizing export and nonoxidative fates over mitochondrial oxidation.^45^ Beyond glycolysis, glutaminolysis provides a significant anabolic lactate source, particularly in some cancer cells, where up to 60% of imported glutamine can be converted to lactate through reductive carboxylation and malic enzyme activity.^44^ In nontransformed cells such as fibroblasts, this flux is markedly lower (∼13%), often supporting acetyl-CoA synthesis rather than export.^46^ Because glutaminolysis-derived lactate originates from mitochondrial carbon skeletons yet is generated cytosolically (via malic enzyme and LDH), it likely favors distinct nonoxidative pools, promoting export, signaling, or biosynthetic routes rather than direct oxidation.

Beyond origin and transport, the chemical form and stereochemistry further constrain fate.

l-Lactate predominantly supports oxidation, signaling, and enzymatic modification, whereas d-lactate, generated through methylglyoxal detoxification and microbial metabolism,^47,48^ is more closely associated with stress-linked modification pathways. Whether d-lactate is pathological or regulated remains open, highlighting stereochemistry as an additional layer of spatial control.

These pathways likely compete for shared lactate pools in a context-dependent manner shaped by transporter activity, LDH localization, redox state, and metabolic flux.^13,18,22,35–37,43^ Under conditions of high lactate flux or limited oxidative capacity, lactate may be increasingly diverted toward signaling or modification pathways.

## 3 Functional Destinations—The Consequences of Subcellular Routing

### 3.1 Destination: Mitochondria—Lactate as an Oxidative Substrate

When lactate is routed to the mitochondrial matrix via mLOC, its dominant fate is oxidation. This routing toward oxidation may limit lactate availability for alternative fates: lactate oxidized to pyruvate and fed into the TCA cycle is less available to be exported for HCAR1 signaling or converted into lactyl donors for protein modification. Substantial experimental evidence supports this route as a major physiological outcome of lactate. In vivo isotopic tracing demonstrates that circulating lactate turns over approximately 2.5-fold faster than glucose and supplies the majority of pyruvate carbon in normal tissues.^43^ This extends to stressed and diseased states where isotope tracers and organ flux studies show that lactate is a dominant oxidative substrate,^49^ and in human lung tumors, it significantly contributes to TCA intermediates, underscoring its role as an oxidative substrate.^50–52^ Consistent with this mitochondrial routing to TCA intermediates, isotope labeling studies in cancer cells (HeLa and H460) show that ∼50% of lactate carbons enter lipids through TCA intermediates such as citrate,^19^ suggesting lactate simultaneously fuels oxidative metabolism while supporting de novo lipid synthesis. In oxidative tissues, this appears to represent a dominant fate of lactate.

The astrocyte-neuron lactate shuttle exemplifies this compartmental access decision at the system level. Glycolytic astrocytes export lactate via MCT4; this lactate is taken up by neurons via MCT1 and oxidized in neuronal mitochondria.^53,54^ The separation of production (astrocyte cytosol) and oxidation (neuron mitochondria) is enforced by cell-type-specific transporter expression and by the mLOC in neurons. Thus, when assessing lactate metabolism in any system, the first question should not be “How much lactate is present?” but “Where is lactate being oxidized?” Increased lactate concentration may indicate increased production, impaired oxidation, or both. The distinction carries very different physiological meanings.

### 3.2 Destination: Extracellular Space and Plasma Membrane—Lactate as a Signaling Molecule

Beyond their metabolic roles, some metabolites directly function as signaling molecules by engaging with receptors or regulatory proteins. It is now clear that metabolites themselves can serve as ligands for G-protein-coupled receptors (GPCRs), linking the metabolic state to signal transduction. Several metabolites act as ligands for Gi-coupled GPCRs, leading to the inhibition of adenylyl cyclase (AC) and reduced cyclic AMP (cAMP) production. Representative examples include adenosine,^55^ short-chain fatty acids,^56^ and lactate.^57,58^

When lactate is routed to the extracellular space rather than retained intracellularly, its primary fate shifts from oxidation to receptor engagement (Figure 3). The Gi-coupled receptor HCAR1 (formerly GPR81) serves as the principal sensor of extracellular lactate.^57–60^ Upon ligand binding, HCAR1 activates Gi, inhibiting adenylyl cyclase, lowering cAMP, and suppressing PKA activity. This signaling axis is especially prominent in adipose tissues, where HCAR1 activation limits phosphorylation of hormone-sensitive lipase and suppresses lipolysis.^8,61,62^ Beyond cAMP signaling, Gi pathways can also signal through βγ subunits to engage ion channels and kinase cascades, including MAPK signaling,^63,64^ broadening the potential impact of extracellular lactate sensing.

**Figure 3:**
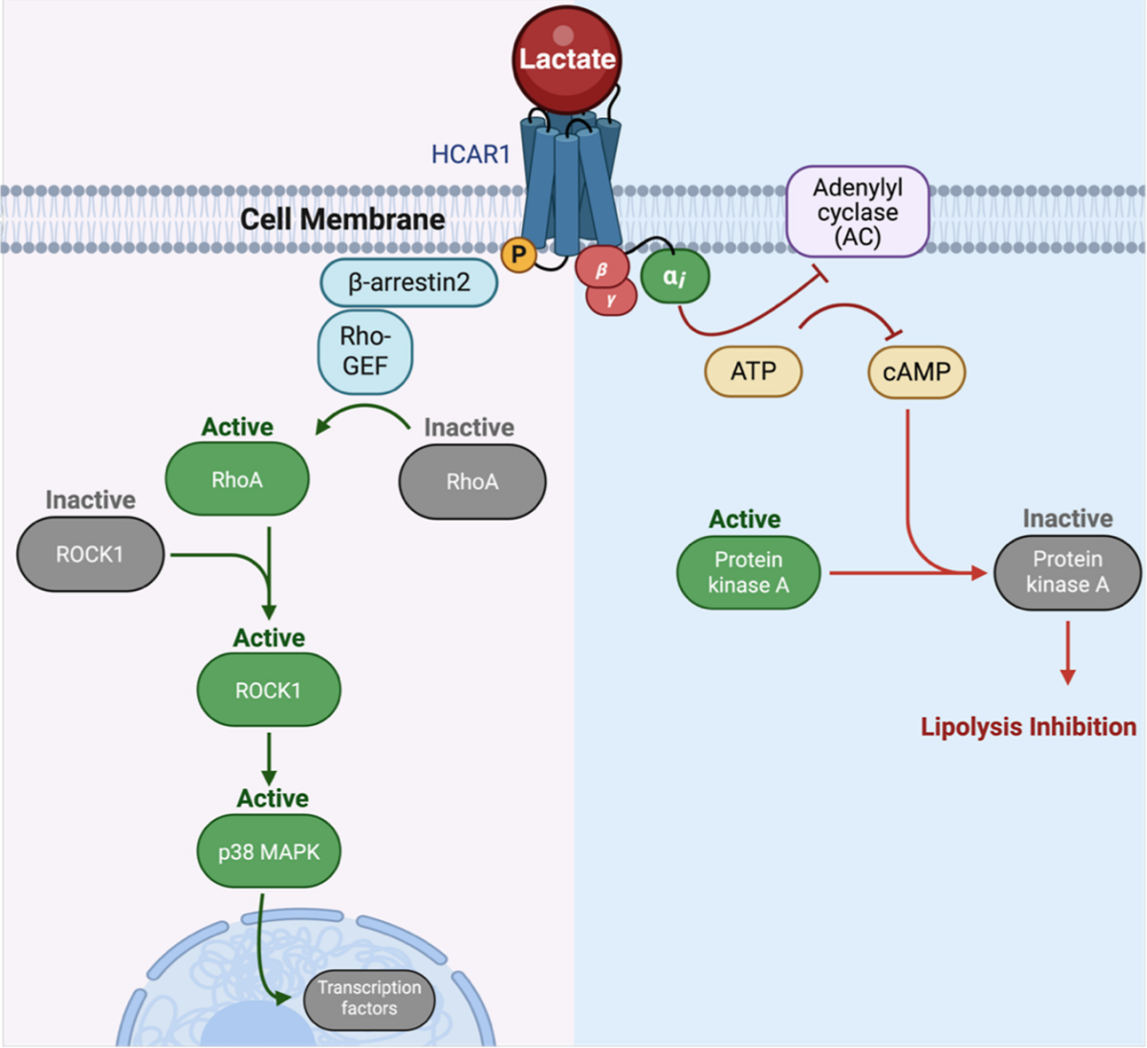
Extracellular routing of lactate to HCAR1 signaling.

Two parallel signaling pathways are activated by lactate binding to HCAR1 at the plasma membrane. Upon activation, Gi dissociates into Gαi and Gβγ subunits. In the canonical pathway (right), Gαi inhibits adenylyl cyclase, reducing cAMP and suppressing protein kinase A activity, ultimately inhibiting lipolysis. In the noncanonical pathway (left), receptor phosphorylation recruits β-arrestin 2, which engages RhoGEF to activate RhoA and ROCK1, driving p38 MAPK activation and downstream transcriptional responses.

In tissues with high HCAR1 expression, particularly adipose tissue, lactate signaling serves an immediate metabolic function by inhibiting lipolysis. Human studies confirm this lipid-sparing effect, with lactate infusion to physiological levels (∼2.7 mM) suppressing postabsorptive lipolysis by approximately 30%, as measured by reduced palmitate flux.^7^ In addition to Gi signaling, β-arrestin and Gβγ-mediated pathways contribute to downstream MAPK activation.

Beyond canonical cAMP signaling, HCAR1 also activates several noncanonical pathways. These include RhoA/ROCK1, p38, β-arrestin 2, and STAT3,^65–67^ though the physiological contexts and functional consequences of these pathways remain incompletely characterized. Notably, a pool of HCAR1 has been localized to the nucleus, where it reportedly activates ERK and AKT and interacts with chromatin remodelers.^5,6^ If confirmed, this emerging finding would represent a fundamentally distinct signaling cascade that requires not only extracellular lactate routing but also receptor trafficking to an intracellular compartment.

In the central nervous system, lactate has been proposed to function as a “volume transmitter”, diffusing through extracellular space to engage HCAR1 on neurons, astrocytes, and endothelial cells; HCAR1 is enriched at the blood–brain barrier and at excitatory postsynaptic membranes.^68,69^ Lactate released from active astrocytes or neurons diffuses through extracellular space to engage HCAR1 on adjacent cells, linking neuronal activity to cerebral blood flow and metabolic support. This signaling axis has been proposed to stabilize neural network activity; age-related memory decline is associated with excessive cAMP and can be rescued by lactate or HCAR1 agonism in animal models.^70–72^

Together, these findings suggest that extracellular lactate participates in a broader sensing network than previously recognized. An open question is whether HCAR1 signaling is purely a readout of extracellular lactate concentration or whether downstream signaling outputs differ depending on lactate’s source (e.g., regulated MCT4 export versus release during injury).

### 3.3 Destination: Nucleus—Lactate as an Epigenetic Substrate

Lactylation illustrates the intersection of routing and chemical form. Similar lysine modifications can arise through distinct donor species, enzymatic pathways, and metabolic contexts, generating modifications that are chemically related but mechanistically distinct. The accumulation of lactate-derived intermediates within nuclear and cytosolic compartments highlights one of lactate’s most direct regulatory roles: serving as a substrate for post-translational modification. The mechanistic basis for this compartmental accumulation, however, remains incompletely resolved. The discovery of protein lactylation^9,10^ provides a mechanistic link between glycolytic flux and the control of chromatin structure and enzymatic activity, linking cellular metabolism directly to proteomic regulation. Historically, histone and protein modifications were thought to be limited to well-known marks such as acetylation, methylation, and phosphorylation, with no role for lactate in epigenetic regulation. Lactylation does not arise solely from elevated lactate levels but from lactate gaining access to specific intracellular compartments and donor-generating enzyme systems. Based on current evidence, metabolic flux appears to be the more dominant determinant under most physiological conditions, while regulated compartmental access becomes critical when distinguishing enzymatic from nonenzymatic lactylation routes.

Mechanistically, these modifications can be divided into enzymatic and nonenzymatic routes. Lactylation is chemically diverse and mechanistically heterogeneous, existing in multiple isoforms, each with different mechanisms and implications. These modifications, collectively termed histone lysine lactylation (HKla), were first identified by mass spectrometry as a +72.021 Da shift on histones. Careful chemical distinction between lactyllysine and carboxyethyl lysine confirmed their unique identities.^9,10^ Overall, three major isoforms have been identified: Lysine d-lactylation (K-d-la) derived from d-lactate metabolism, l-lactylation (K-l-la) driven by l-lactate, and lysine carboxyethylation (Kce), a nonenzymatic glycation-like adduct formed under stress. Unlike acetylation, lactylation is highly context dependent, varying with metabolic flux, cellular stress, and lactate stereochemistry. These distinct chemistries likely emerge from different routing environments: enzymatic lactylation arises from regulated nuclear or cytosolic access to activated lactyl donors, whereas nonenzymatic adducts accumulate when lactate is diverted into stress-associated metabolic pathways (Figure 4).

**Figure 4:**
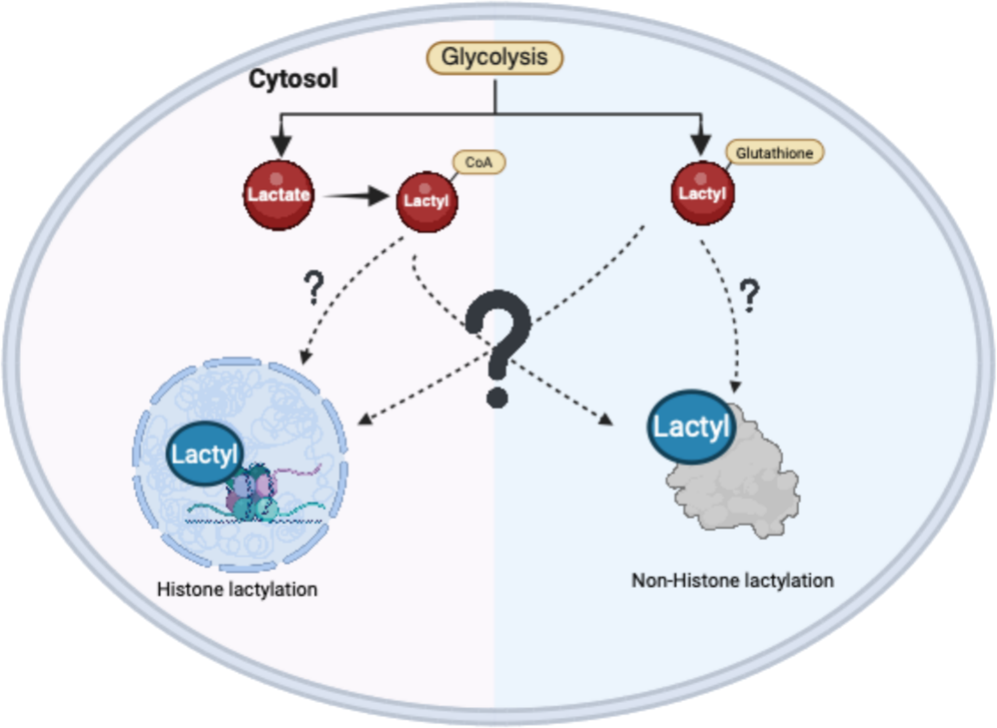
Multiple lactyl donor pathways link lactate to protein modification; glycolysis-derived lactate can enter two alternative donor pathways. In one route, lactate is converted into lactyl-CoA, a proposed donor for enzymatic histone lactylation. In the second, methylglyoxal metabolism generates lactoylglutathione (LGSH), which may contribute to nonenzymatic protein modification and d-lactate production. Multiple donor species may contribute to lactate-associated protein modifications. The relative contribution of lactyl-CoA, lactoyl-glutathione, d-lactate-derived intermediates, and other donor pools remains an active area of investigation. Importantly, these pathways are not equivalent; lactyl-CoA is proposed to support regulated enzymatic lactylation, whereas lactoyl-glutathione is linked primarily to nonenzymatic modification and methylglyoxal detoxification.

Under conditions of high glycolytic flux, lactate may be preferentially diverted into enzyme-driven modification pathways. Current models propose that at least some enzymatic lactylation proceeds via a lactyl-CoA intermediate, in which Coenzyme A functions as a leaving group during nucleophilic attack by the ε-amino group of lysine.^73^ Although lactyl-CoA is detectable at low abundance,^74^ its functional relevance is supported by the observation that several histone acetyltransferases moonlight as lactyltransferases. Identified writers include proteins such as p300, General control nondepressible 5 (GCN5) (catalyzing H3K18la during cardiac injury), Histone Acetyltransferase Binding to ORC1 (HBO1) (targeting H3K9la), Alanyl-tRNA synthetase 1 (AARS1/2) in tumor-associated lactylation,^10,21,75^ as well as Lysine Acetyltransferase 5/Tat-interactive protein 60 kDa (KAT5/TIP60)^76^ and Lysine Acetyltransferase 8 (KAT8).^77^ In glioma, the nuclear GTP-specific Succinyl-CoA Synthetase (GTPSCS) complex preferentially synthesizes lactyl-CoA over succinyl-CoA and cooperates with p300 to promote H3K18la,^20^ supporting the existence of a compartmentalized nuclear lactylation machinery. The glyoxalase pathway generates LGSH as part of a protective response to methylglyoxal accumulation and represents an alternative mechanism through which lactate-derived intermediates modify proteins via nonenzymatic mechanisms.

Not all lactylations are enzyme-mediated. LGSH formation via the glyoxalase system represents a detoxification pathway for methylglyoxal (MG), ultimately yielding d-lactate. In contrast, lysine carboxyethylation (Kce) arises from nonenzymatic reactions of MG with proteins and is generally considered a stress-associated modification. Thus, LGSH formation can be viewed as a protective mechanism that limits MG accumulation, whereas Kce reflects incomplete detoxification under conditions of an elevated MG. Although Kce and lysine lactylation share identical mass shifts and can increase in parallel, Kce is chemically distinct and should not be considered a direct lactate-derived modification.^78^

As with other acyl modifications, lactylation likely operates within a writer–eraser–reader framework. Several acyltransferases have been identified as writers; however, dedicated erasers remain undefined, and bona fide reader domains specific for lactyl-lysine have not been established. Candidate reader-like interactions have been reported, including Brahma-related gene-1 (Brg1) (Smarca4) binding to H3K18la during induced pluripotent stem cell reprogramming^79^ and Double PHD Fingers 2 (DPF2) colocalization with H2K14la at oncogenic promoters.^80^ Yet no canonical bromodomain, YEATS, or Plant Homeodomain (PHD) domain has been validated to selectively recognize lactylation, leaving a major mechanistic gap in the field.

Within this spatial logic, the identity of the lactyl donor is likely compartment-dependent, determined by where lactate accumulates and which biochemical environment enables its activation to reactive intermediates. A central unresolved question is the identity of the dominant lactyl donor in vivo. Competing models distinguish between a regulated acyl-transfer pathway driven by lactyl-CoA and a stress-linked thioester relay mediated by lactoyl-glutathione. Resolving which donor predominates under defined physiological conditions is essential to understanding how lactate routing governs its epigenetic function.

### 3.4 Destination: Cytosol—Lactate as an Endocrine Precursor

When lactate is routed through the cytosolic enzyme carnosine dipeptidase 2 (CNDP2), it is conjugated to amino acids to form N-lactoyl-amino acids (N-Lac-AAs).^12^ This pathway diverts lactate toward an endocrine signaling fate rather than oxidation or local-receptor signaling. Here, the resulting N-Lac-AAs, including N-lactoyl-Phe (Lac-Phe), N-lactoyl-(iso)­Leu, N-lactoyl-Tyr, and N-lactoyl-Trp, are then exported from the cell by the ATP-binding cassette subfamily C member 5 (ABCC5) transporter into systemic circulation.^11^ The mechanism of lactyl donation to CNDP2, whether it uses free lactate or requires an activated intermediate, has not yet been resolved. Although CNDP2 activity was initially characterized in metabolic tissues with high glycolytic flux, this pathway is not restricted to these contexts, indicating that N-lactoyl amino acid production may occur across diverse cellular contexts. Recent work reveals that microglia express both CNDP2 and the lactate transporter MCT1, identifying them as a local cellular source for Lac-Phe production in the brain, particularly in response to high-calorie diet intake.^81^ While Lac-Phe is the best-characterized member of this class, other N-Lac AAs remain speculative, and their inclusion here is intended to illustrate principle rather than completeness.

Physiologically, during exercise, circulating Lac-Phe rises sharply, especially in individuals with obesity, consistent with high lactate flux driving its synthesis.^12^ Once exported into circulation, it suppresses food intake by activating KATP channels on hypothalamic Agouti-related protein (AgRP) neurons, reducing hunger signaling.^12,82^ This system also responds to the pharmacological perturbation. Pharmacologically, metformin stimulates Lac-Phe production by inhibiting complex I and enhancing glycolytic lactate flux; CNDP2 deletion blunts metformin-induced weight loss in both mice and humans.^83,84^ Thus, the physiological effects of Lac-Phe arise not from lactate accumulation alone but from its diversion into a specific cytosolic biosynthetic and export pathway. This provides a clear example in which a lactate routing pathway can be traced from the molecular mechanism to the organismal phenotype.

Beyond Lac-Phe, N-lactoyl-tryptophan is a potent taste-modifying molecule,^85^ suggesting that different N-Lac-AAs may have unique biological functions. Additional N-lactoyl metabolites likely remain to be defined. This pathway requires cytosolic lactate retention and access to the CNDP2. It is a competitive routing decision; activation of this pathway diverts lactate away from both the MCT4 export and mLOC import. Understanding what determines this prioritization, including which cell types preferentially engage CNDP2 under what metabolic conditions, remains a key open question.

## 4 Integrative Model, Testable Predictions, and Future Directions

We propose a broader shift in metabolic understanding: lactate is neither a waste product nor an indiscriminately multifunctional metabolite but a versatile intermediate whose specific role is determined by subcellular routing.

This spatial organization generates several testable predictions:

1. Inhibition of mitochondrial lactate import should increase the availability for nuclear or cytosolic modification.
2. Enhanced CNDP2 activity may reduce the oxidative lactate flux while also limiting lactate availability for export and local signaling.
3. Manipulation of the MCT1/MCT4 expression should alter the balance between oxidation and extracellular signaling.
4. Compartment-targeted lactate biosensors should reveal whether nonequilibrium lactate gradients exist between the cytosol, nucleus, and mitochondria.
5. Manipulations that alter lactate compartmentalization should produce larger changes in the biological outcome than equivalent manipulations that alter bulk cellular lactate abundance.

Critical questions remain, including mechanisms controlling lactate trafficking between compartments, the identity of the dominant lactyl donor in vivo, and the consequences of disrupted routing in cancer and metabolic diseases. Addressing these will require compartment-specific biosensors and spatial metabolomics capable of resolving the lactate flux with precision.

A major implication of this framework is that bulk lactate measurements alone may be insufficient to infer the biological function. Similar bulk lactate concentrations may reflect fundamentally different metabolic states, including mitochondrial oxidation, extracellular signaling, and epigenetic modification. Resolving these distinctions will require experimental approaches capable of measuring compartment-specific lactate flux rather than the total abundance alone. Importantly, we do not propose that all lactate functions require strict compartmental gating; rather, we suggest that spatial organization biases lactate utilization toward distinct biological outcomes.

More broadly, the principle emerging from lactate biology may extend beyond lactate itself. The concept that a molecule’s function is defined by its cellular address and chemical identity may represent a fundamental principle of metabolic organization, applicable to succinate, acetyl-CoA, and other multifunctional intermediates. Viewed this way, lactate reveals an additional layer of metabolic regulation in which cellular location helps shape biological function. In this framework, lactate is not multifunctional simply because it performs many roles simultaneously, but because distinct routes and chemical forms direct it into biochemical contexts that define its biological role.

### 4.1 Limitations and Outstanding Questions

Despite increasing evidence supporting compartmentalized lactate metabolism, several key uncertainties remain. Direct measurements of subcellular lactate concentrations, particularly within the nucleus, have been limited. The identity and relative contribution of lactyl-donor species, including lactyl-CoA and LGSH, are still incompletely resolved. The regulation, tissue specificity, and physiological relevance of mitochondrial lactate oxidation also require further investigation. Additionally, the extent to which lactate routing reflects actively regulated transport versus emergent properties of concentration gradients, enzyme localization, and metabolic kinetics remains unresolved. Addressing these gaps will require improved tools for compartment-specific metabolite measurement and flux analysis.^43^ Beyond the mechanisms discussed here, the routing framework proposed in this Perspective is unlikely to be exhaustive. Additional lactate functions may arise through direct molecular interactions that depend on local concentration rather than subcellular destination.^86^ Thus, compartmental routing should be viewed as a unifying organizational principle rather than as a complete accounting of all possible lactate-mediated effects. Future studies that directly measure compartment-specific lactate flux rather than total lactate abundance will be essential for determining how broadly this principle applies across physiology and disease.
